# In Silico Analysis of the Association Relationship between Neuroprotection and Flavors of Traditional Chinese Medicine Based on the mGluRs

**DOI:** 10.3390/ijms19010163

**Published:** 2018-01-10

**Authors:** Xu Zhang, Liansheng Qiao, Yankun Chen, Bowen Zhao, Yu Gu, Xiaoqian Huo, Yanling Zhang, Gongyu Li

**Affiliations:** Key Laboratory of TCM-Information Engineer of State Administration of TCM, School of Chinese Pharmacy, Beijing University of Chinese Medicine, Beijing 100102, China; 18003381008@163.com (X.Z.); b20100222012@163.com (L.Q.); 18811791975@163.com (Y.C.); zhaobw9504@163.com (B.Z.); riberyguyu@163.com (Y.G.); aixue_126@126.com (X.H.); lidoc2727@163.com (G.L.)

**Keywords:** metabotropic glutamate receptors (mGluRs), traditional Chinese medicine (TCM), five flavors, neuroprotection, allosteric, virtual screening

## Abstract

The metabotropic glutamate receptors (mGluRs) are known as both synaptic receptors and taste receptors. This feature is highly similar to the Property and Flavor theory of Traditional Chinese medicine (TCM), which has the pharmacological effect and flavor. In this study, six ligand based pharmacophore (LBP) models, seven homology modeling models, and fourteen molecular docking models of mGluRs were built based on orthosteric and allosteric sites to screening potential compounds from Traditional Chinese Medicine Database (TCMD). Based on the Pharmacopoeia of the People’s Republic of China, TCMs of compounds and their flavors were traced and listed. According to the tracing result, we found that the TCMs of the compounds which bound to orthosteric sites of mGluRs are highly correlated to a sweet flavor, while the allosteric site corresponds to a bitter flavor. Meanwhile, the pharmacological effects of TCMs with highly frequent flavors were further analyzed. We found that those TCMs play a neuroprotective role through the efficiencies of detumescence, promoting blood circulation, analgesic effect, and so on. This study provides a guide for developing new neuroprotective drugs from TCMs which target mGluRs. Moreover, it is the first study to present a novel approach to discuss the association relationship between flavor and the neuroprotective mechanism of TCM based on mGluRs.

## 1. Introduction

The organoleptic characteristics of traditional medicine, especially smells and flavors, reveal significant information about their curative effect [1,2]. People around the world have different cognitive information for organoleptic characteristics. To be specific, people in Northeastern Brazil classified the taste into five types, including bitter taste, good taste, no taste, bad taste, and sweet taste [2]. Taste is classified into four types—sweet, spicy, acidic, and harsh (astringent)—in northwestward Patagonia [3]. Similarly, as a typical traditional medicine, five flavors are also proposed based on the theory of five flavors from traditional Chinese medicine (TCM), including sour, bitter, sweet, pungent, and salty. At the same time, five flavors are also highly related to the traditional pharmacological effect of TCM based on the clinical experience. On the other hand, according to modern medical studies, the correlation between the theories of five flavors and Chinese medicine pharmacology has been proven to some extent. For instance, herbal medicines with bitter flavor have a better therapeutic effect for diabetes mellitus [4]. According to data mining, sweet or bitter herbs appeared with high frequency in the Chinese medicinal prescription of breast cancer recurrence and metastasis [5]. However, the relationship between modern pharmacological effect on TCM and its five flavors is still not clear. 

In the oral cavity, the taste receptor is devoted to detect compounds with gustatory stimulation in foodstuffs and transmit their signals to gustatory nerve fibers. Salty and sour tastes are mediated by channel type receptors, while sweet, bitter, and umami tastes are mediated by G-protein coupled receptors (GPCRs) and second messenger signaling cascades [6]. The metabotropic glutamate receptors (mGluRs), as a member of the GPCR family, are umami taste receptors [7]. In this paper, mGluRs were introduced to discuss the association relationship among taste receptors, flavors, and pharmacological effect of TCMs. Based on sequence homology, the mechanisms of intracellular signaling and pharmacological characters, mGluRs are divided into three clusters. The group І mGluRs (mGluR І) were mainly distributed in the postsynaptic membrane consisting of mGluR1 and mGluR5. The groups ІІ and ІІІ mGluRs (mGluR ІІ, mGluR ІІІ) were mainly distributed in the presynaptic membrane. mGluR ІІ is composed of mGluR2 and mGluR3, and mGluR ІІІ consists of mGluR4, mGluR6, mGluR7, and mGluR8 [8,9]. However, it is worth mentioning that all mGluRs subtypes are found in the cerebellar cortex, with the exception of mGluR6, which is exclusively expressed in the retina and not within the scope of our research. As the typical GPCRs, mGluRs with the structure of seven transmembrane domain (7TMD) have two distinct sites. The orthosteric site was distributed on the extracellular domains of mGluRs, and the allosteric site was found in the transmembrane domains [10]. The mGluRs, which serve as the main excitatory neurotransmitter in the mammalian central nervous system (CNS), play an important role in many basic brain functions such as learning and memory, neuroendocrine regulation, and emotional homeostasis. Research shows that mGluR І are considered as promising therapeutic targets of depression, anxiety, chronic pain and Alzheimer’s disease [11]. The mGluR ІІ and mGluR ІІІ are involved in the process of epilepsy, Parkinson’s disease, and so on [12]. Antagonists and negative allosteric modulators (NAMs) of mGluR І, and agonists and positive allosteric modulators (PAMs) of mGluR ІІ and mGluR ІІІ have neuroprotective effects. In addition, TCM has a wide range of applications in neuroprotection. Thus, corresponding relationships between flavors and the neuroprotective mechanism of TCM might be interpreted to some extent based on mGluRs.

**Figure 1:**
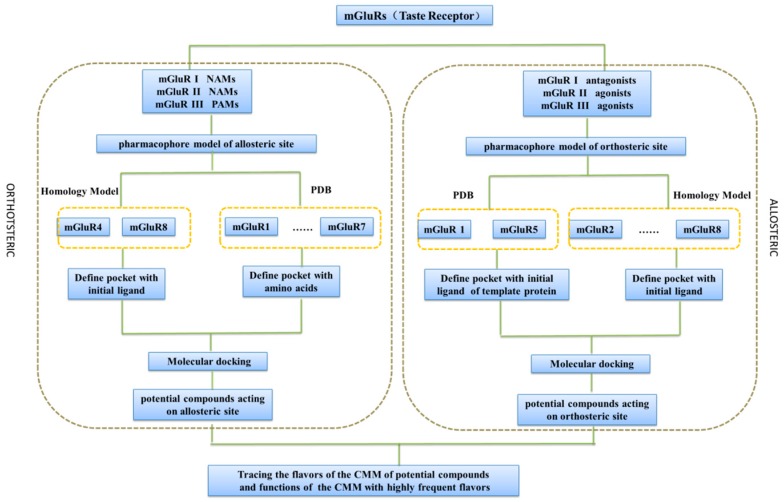
The strategy of analyzing the association relationship between flavor and neuroprotection of traditional Chinese medication (TCM) based on the metabotropic glutamate receptors (mGluRs).

In this paper, antagonists and negative allosteric modulators (NAMs) of mGluR І, and agonists and positive allosteric modulators (PAMs) of mGluR І and mGluR ІІІ were collected to generate orthosteric and allosteric pharmacophore models of mGluRs, respectively. Then, homology modeling and molecular docking was also used in combination to discover the potential compounds acting with mGluRs respectively. Besides this, the flavors and efficacies of TCM of those potential compounds were traced to interpret the neuroprotective mechanism of TCM to some extent. The strategy of this study was shown in Figure 1.

## 2. Results

### 2.1. Pharmacophore Model Studies

The orthosteric antagonists and NAMs of mGluRI as well as the orthosteric agonists and PAMs of mGluR ІІ and mGluR ІІІ were collected from the literature, respectively [8,13,14,15,16]. After removing the duplicated compounds, orthosteric and allosteric compounds of three groups of mGluRs were retained. Based on the above compounds, six orthosteric antagonists and eight NAMs of mGluR I, seven agonists and seven PAMs of mGluR II, as well as eight agonists and six PAMs of mGluR III were selected to build pharmacophore hypotheses, respectively. The chemical information of the above compounds was shown in Figure S1, respectively.

Intrinsic parameters such as Specificity, N_hits, Pareto, Energy, Sterics, Hbond, and Mol_qry were used to evaluate the generated models of each group. The best models of pharmacophore model of each group were selected based on three criteria [17,18]: (1) The number of “hits” (N_hits) and the number of compounds which were used to generate the pharmacophore models ought to be approximately equal; (2) The model has lower Energy and higher Specificity, Sterics, Hbond, and Mol_qry; (3) The Pareto values should be zero, which indicates that the generation of the model is not accidental. Finally, ∑Ranking was used to evaluate all indicators synthetically, and the model which possesses the lowest ∑Ranking value was regarded as the optimal model [19].

**Figure 2:**
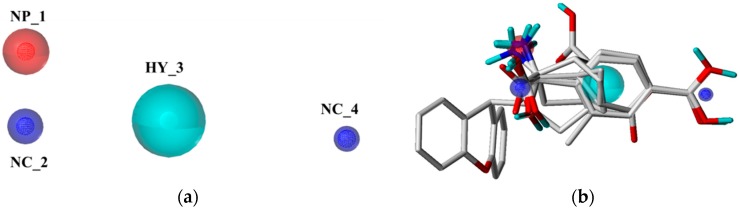
(**a**) The optimal pharmacophore model of orthosteric site of mGluR I (model 03); (**b**) Model 03 mapped with the compound used to build model.

**Figure 3:**
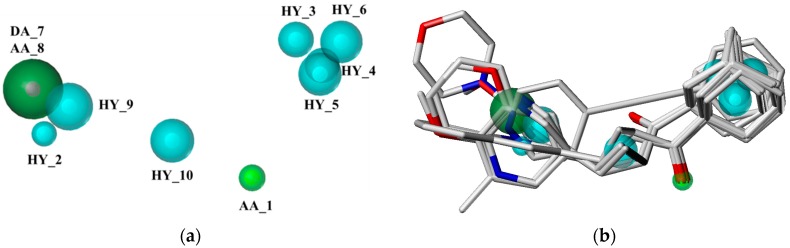
(**a**) The optimal pharmacophore model of allosteric site of mGluR I (model 01); (**b**) Model 01 mapped with the compound used to build model.

Taking the pharmacophore of orthosteric antagonists of mGluR I as an example, the pharmacophore models were generated based on the six compounds, and the nine models with N_hits value of 6 were chosen and displayed in Table 1. It indicates that the selected models are mapped all of the compounds which used to generate the pharmacophore models. As can be seen from the table, the Pareto ranks of the remained models were “0”, which implies that models were not superior to each other. Then, ranking values were assigned based on the observations of the five factors (Specificity, Energy, Sterics, Hbond, and Mol_qry). The model with the lower Energy was designated as number 1. On the contrary, for the other four intrinsic parameters, the model which possesses the largest values were designated the number 1. The results of ∑Ranking were shown in Table 2, and model 3 with smallest value of ∑Ranking was chosen as the best pharmacophore model (Figure 2) to screen potential mGluR I antagonists from the Traditional Chinese Medicine Database (TCMD). It contained four features, including one positive nitrogen (NP_1), one hydrophobic feature (HY_3), and two negative centers (NC_2, NC_4). Similarly, for the allosteric pharmacophore of mGluR I, the best model was selected and shown in Figure 3. The optimal model selection results of other groups are shown in Figure S2. The results of the remaining pharmacophores were shown in Tables S1–S4.

### 2.2. Database Search

The six optimal pharmacophore models of orthosteric and allosteric sites of three groups of mGluRs were used as 3D queries to search TCMD, respectively. The screening of the six pharmacophore queries yielded 123,178,110 potential orthosteric compounds and 320, 532, and 1714 potential allosteric compounds of mGluRs I, II, and III respectively. 

Then the hit compounds were filtered based on “Lipinski’s rule of five” (≥4) [20]. In this case, 89,103 and 80 potential drug-like orthosteric compounds were obtained as well as 221,287 and 392 potential drug-like allosteric compounds. However, choosing all these natural compounds for the next study was not a wise strategy, as only parts of compounds are able to cross the blood-brain barrier. Therefore, the prediction research on blood–brain barrier permeability of the candidate compounds was implemented by ADMET module within Accelrys Discovery studio 4.0 [19]. Those compounds which were predicted to cross the blood–brain barrier were retained and optimized. The results of pharmacophore screening are summarized in Table 3. 

### 2.3. Homology Modeling Studies

For the mGluRs family, several crystal structures of extracellular domains containing orthosteric binding sites have been resolved, including mGluR1, mGluR2, mGluR3, mGluR5, and mGluR7. However, only three crystal structures of 7TMD, which contains the allosteric binding site, have been resolved, including mGluR1, mGluR5, and mGluR7. Therefore, the extracellular domains of mGluR4 and mGluR8 were generated by homology modeling through the MODELLER program, as well as the 7TMD domain of mGluR2, mGluR3, mGluR4, mGluR7, and mGluR8.

From the BLAST results, the structure of mGluR7 (PDB ID: 3MQ4, resolution, 2.8 Å), which possesses the highest sequence identity value, was chosen as template protein of the extracellular domains of mGluR4 and mGluR8 to build Homology modeling models. Similarly, structure of mGluR1 (PDB ID: 4OR2, resolution, 2.8 Å), mGluR5 (PDB ID: 4OO9, resolution, 2.6 Å) and mGluR5 (PDB ID: 5CGC, resolution, 3.1 Å) were respectively selected as template proteins of 7TMD of mGluR4, mGluR7, and mGlur8 (Table 4).

Then, homology modeling models of mGluRs were constructed by the MODELLER program. Next, structural refinement was performed by the conformational sampling using molecular dynamics (MD) simulation. The conformation with the lowest energy was selected as the optimal model for each mGluRs and then performed the energy minimization. Two indicators, Ramachandran plot and ERRAT, were used to evaluate the models [21]. The results of the evaluation were displayed in Table 4. The results of Ramachandran plot showed that the residues of each model located in the allowed regions were greater than 90%, and the views of the ERRAT score, overall quality factors were greater than 80. The results of Ramachandran plot and ERRAT suggested that all models of mGluRs could be applied for future studies. The homology modeling models and the Ramachandran plots are shown in Figures S3 and S4.

### 2.4. Molecular Docking Studies

The molecular docking algorithm of Surflex-Dock was used to further refine the pharmacophore screening results. Surflex-Dock is a fully automatic flexible molecular docking algorithm that combines the scoring function from the Hammerhead docking system with a search engine that relies on a surface-based molecular similarity method as a means to rapidly generate suitable putative poses for molecular fragments [22,23]. It implements a progressive building search approach, as in Hammerhead, and implements a faster and more accurate method of assembly of fragments [22]. 

#### 2.4.1. The mGluRs Orthosteric Site

For the mGluR1, mGluR5, mGluR2, mGluR3, and mGluR7, the crystal structure has been resolved. The active pockets were defined by the location of the ligand in the crystal structure. The structure of mGluR4 and mGluR8 were built by homology modeling using 3MQ4 (mGluR7-LY341495) as a template. LY341494 (Z99) is a competitive antagonist of mGluR III. The active pockets of mGluR4 and mGluR8 were defined by the location of LY341494 in 3MQ4.

**Figure 4:**
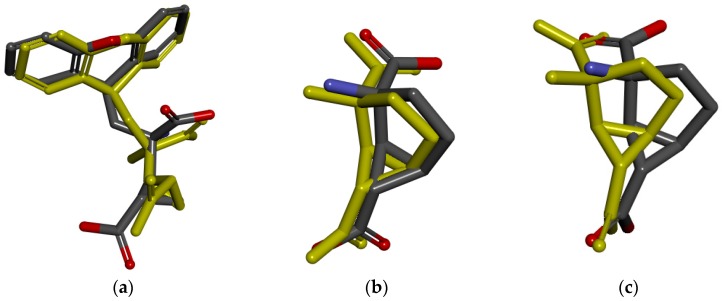
The comparison between the initial binding pose (yellow) and the docked pose. (**a**) mGluR1; (**b**) mGluR2; (**c**) mGluR3.

Then, different methods are implemented in order to verify the reliability of the docking algorithm and docking parameters. For the mGluR1, mGluR2, and mGluR3, after being extracted from the orthosteric binding site, the initial compounds were re-docked into the crystal structure, and then the RMSD values between docked pose and initial binding pose were 1.4946 Å, 0.5063 Å, and 1.1403 Å (Figure 4), which indicated that the selecting of docking algorithm Surflex-Dock and setting of docking parameters were reasonable. For the docking models of mGluR4, mGluR7, and mGluR8, the crystal structure complexed with competitive antagonists, but the compound we need to virtual screen is an agonist. The mGluR5 has a similar problem. Therefore, it is not suitable to verify the rationality of the active pocket by comparing the RMSD value. The known binders of mGluR4, mGluR5, mGluR7, and mGluR8, collected separately from the literature [24], were docked into the corresponding pockets, respectively. Then, the correlation between the rank of compounds experimental affinity (Ki) and docking score were calculated. The correlations of GluR4, mGluR7, and mGluR8 were 0.9218, 1, and 0.8167, respectively (Table 5, Table 6 and Table 7). It is worth mentioning that mGluR5 known binders lack of affinity data, and correlation cannot be calculated. However, all known binders were successfully docked into the pockets with the total score more than four [25,26], which suggested that docking method selection and parameter setting are reasonable is applicable. The details of identification and evaluation of the orthosteric active sites are shown in Table 8. 

**Figure 5:**
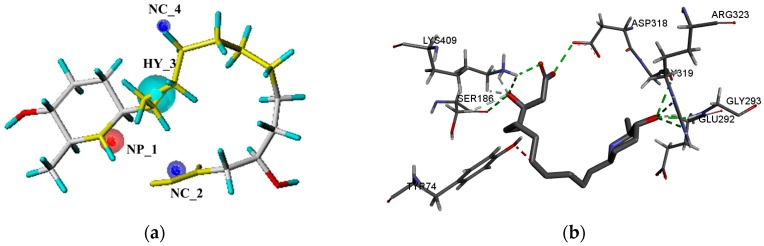
(**a**) The mapping results of Morusimic acid D with the best pharmacophore of orhtosteric site of mGluR I; (**b**) the docking result of Morusimic acid D with the crystal mGluR1.

Next, the compounds which were screened by six optimal pharmacophore models were docked into the corresponding seven orthosteric active pockets of mGluRs, respectively. Seven sets of candidate compounds were obtained. The mGluR1 was taken as an example to analyze the results of molecular docking. For molecular docking of orthosteric site, the initial ligand (Z99) could form hydrogen bond interactions with ARG78, SER165, SER186, THR188, and LYS409(Figure 5b). The interaction between potential active compounds and crystal structure of mGluR1 was also analyzed, such as Morusimic acid D which is isolated from *Morus alba*. Same as the initial ligand, Morusimic acid D could form hydrogen bond interactions at the orthosteric site of mGluR1 with the amino acids SER186 and LYS409. Then, the accuracy of pharmacophore and docking model were further analyzed. The pharmacophore results indicated that Morusimic acid D could match with three features of the optimal pharmacophore model (Figure 5a). Furthermore, the hydroxyl group in the carboxyl group could form hydrogen effect with SER165 and also map with negative ionizable group (NC_2) in pharmacophore. Therefore, the results showed the consistency of docking and pharmacophore model. Matching maps of pharmacophore model and molecular docking model of representative potential candidate compounds acting on the allosteric sites for the remaining mGluR subtypes were listed in Figures S5–S10.

#### 2.4.2. The mGluRs Allosteric Site

For mGluR1 and mGluR5, the active pockets were defined by the initial ligands, respectively. The active pockets of remaining models which obtained through homology modeling were defined by amino acids [27]. 

**Figure 6:**
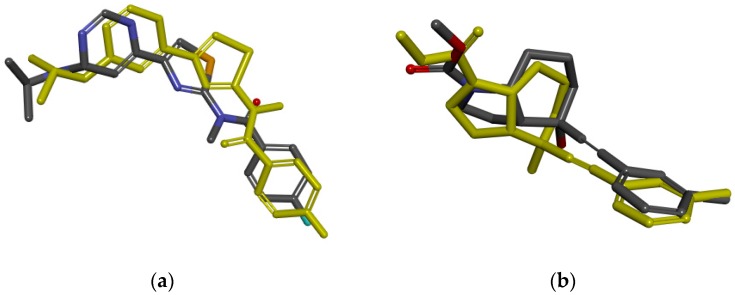
The comparison between the initial binding pose (yellow) and the docked pose. (**a**) mGluR1; (**b**) mGluR5.

Similar to orthosteric active pockets, the initial compounds of mGluR1 and mGluR5 were extracted and re-docked into the crystal structure, and the RMSD values among five re-docked ligands and crystal structures were calculated, which suggested the rationality of docking algorithm Surflex-Dock and docking parameters. The comparisions between the initial binding pose interactions and the docked pose interactions of mGluR1 and mGluR5 are presented in Figure 6. The remaining molecular docking models, because of the lack of experimental affinity values (Ki or Kd) of the known binders and absence of ligand of crystal structures, RMSD values between docked pose and initial binding pose or correlation between the rank of compounds experimental affinity (Ki) and docking score cannot be computed. Nevertheless, the known binders were successfully docked into the corresponding pockets. All the total scores were more than four, which shows that docking methods selection and parameter settings are reasonable [25,26]. The details of identification and evaluation of the allosteric active pocket are shown in Table 9.

Next, the compounds screened by the optimal pharmacophore of mGluRs allosteric sites were docked into the corresponding pockets, respectively. Finally, allosteric candidate compounds of seven subtypes of mGluRs with a total score more than four were obtained. 

**Figure 7:**
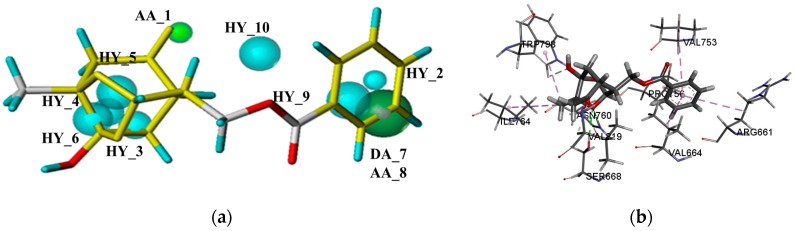
(**a**) The mapping results of Paeoniflorigenone with the best pharmacophore of allosteric site of mGluR І; (**b**) the docking result of Paeoniflorigenone with the crystal structure of mGluR1.

The mGluR1 was taken an example to demonstrate the analysis of docking results. According to the previous study, NAMs of mGluR1 can form hydrogen bond interactions with THR794, THR815, and SER822, and form hydrophobic interactions with PRO756, LEU757, and TRP798. Then, the interaction between potential allosteric active compounds and the transmembrane domain of mGluR1 were analyzed. Paeoniflorigenone is an ingredient which was isolated from *Paeonia lactiflora*. Same as the initial ligand, it can generate hydrophobic interactions with PRO756 and TRP798. Besides, Paeoniflorigenone can also form hydrogen bond with ASN760 (Figure 7), which was also been identified as the key residue for mGluR1 NAMs by Fukuda [11]. Then, the results of pharmacophore and molecular docking were discussed comprehensively to further analyze its accuracy. The aromatic rings of the compound which could produce hydrophobic effect with mGluR1 were also mapped with the corresponding hydrophobic features (HY_2 and HY_9). The oxygen atom of the hydroxyl group could perform the hydrogen bond with mGluR1, and mapped with hydrogen features (AA_1) in pharmacophore. On the basis of the above analysis, the results of pharmacophore and molecular docking are consistent. Pharmacophore mapping results and molecular docking results of representative potential candidate compounds acting on the allosteric sites for the remaining mGluR subtypes are listed in Figures S11–S16.

### 2.5. Data Analysis

According to Chinese Pharmacopoeia (2015 version), the TCMs and their flavors were traced based on the potential compounds acting on orthosteric and allosteric sites of mGluR І, mGluR ІІ, and mGluR ІІІ. Six sets were listed in Tables S5–S10. 

Interestingly, we found that the TCMs of the compounds which bound to orthosteric site are highly correlated to sweet flavor. To be specific, the TCMs corresponding to mGluR І mostly possess sweet flavor and bitter flavor; mGluR ІІ is relevant to sweet flavor and pungent flavor; and mGluR ІІІ is only related to sweet flavor. Moreover, numerous TCMs of the potential compounds have been proven to have an active effect on neuroprotection, including *Fructus Lycii (Gou Qi Zi)* [28], *Semen Gingko (Bai Guo)* [29], *Fructus Mori (Sang Shen)* [30], *Juncus effuses (Deng Xin Cao)* [31], *Semen Trigonellae (Hu Lu Ba)* [32], *Angelica sinensis (Dang Gui)* [33], *Litchi chinensis Sonn (Li Zhi He)* [34], and *Apis cerana (Feng Mi)* [35]. *Fructus Lycii* [28], *Angelica sinensis* [33], and *Litchi chinensis Sonn* [34] have been shown to have neuroprotective potential for treating Alzheimer’s disease or other aging-related neurodegenerative diseases. Besides this, *Semen Gingko* [29] can treat ischemic brain injury, as well as *Fructus Mori* [30]. *Semen Gingko* [29], *Semen Trigonellae* [32], and *Apis cerana* [35] have been reported to protect neurons against oxidative stress. In addtion, *Juncus effuses* [31] is traditionally used as sedative and to treat health problems like insomnia.

On the contrary, the TCMs of the compounds which bound to allosteric site are highly correlated with bitter flavor. More specifically, the TCMs corresponding to mGluR І and mGluR ІІІ mostly possess bitter flavor and pungent flavor; the mGluR ІІ is relevant to bitter flavor. Many TCMs have been proven to be effective Chinese medicines to treat neurological diseases, including *Corrydalis yanhusuo (Yan Hu Suo)* [36], *Psoralea corylifolia Linn (Bu Gu Zhi)* [37], and *Radix Paeoniae Alba (Bai Shao)* [38]. Specifically, *Corrydalis yanhusuo* [36] and *Psoralea corylifolia Linn* [37] play neuroprotective effects through treatment of dementia and oxidative stress, respectively. In addition, *Radix Paeoniae Alba* [38] could treat Parkinson’s disease by reducing the MPTP-induced toxicity. To some extent, the content mentioned above proved the reliability of the screening results of present study. 

Besides this, traditional pharmacological effects of the TCMs which correspond to the highly frequent flavors were traced. According to occurrence frequency, the effects were arranged from high to low. The top-ranked functions of each group were shown in Table S11. Based on the table, the three most representative functions of each group were selected.

**Figure 8:**
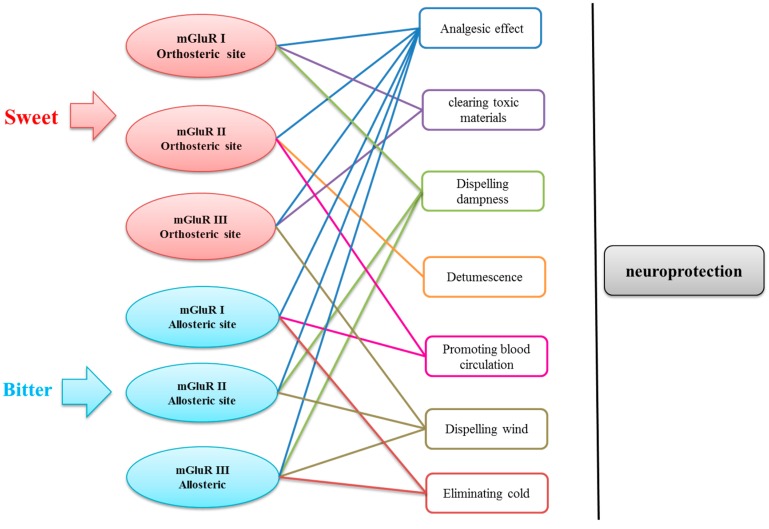
The sketch map of flavors, targets, and efficiencies.

The relationships between target–five flavors–functions were summarized and shown in Figure 8. As can be seen from the figure analysis, TCMs of compounds which bound to orthosteric site of mGluRs mainly possess sweet flavor, whereas TCMs of compounds which acting on allosteric site of mGluRs mainly have bitter flavor. The three most representative functions of each group were displayed and connected corresponding targets with straight line. In general, TCMs with different acting on mGluRs could produce similar neuroprotective effect. For example, *Angelica sinensis* with sweet flavor contains potential compounds acting on orthosteric site of mGluR ІІ. It has functions of analgesic effect and promoting blood circulation, and *Angelica sinensis* has been shown to have the neuroprotective effect of treating Alzheimer’s disease and vascular dementia [33]. Therefore, it is found that the result of the study is reliable to some extent.

## 3. Materials and Methods

### 3.1. GALAHAD Pharmacophore Hypotheses Generation

The building process of the GALAHAD model consists of two steps [39,40]. First, all the compounds were aligned each other in intrinsic coordinate space. During this process, a genetic algorithm (GA) was operated to identify a set of conformations of compounds with low strain energy (SE), steric overlap (SO), and pharmacophoric similarity (PhS). Second, the optimal set of conformations were aligned in Cartesian space as rigid-bodies. In this step, geometric heuristics and linear assignment methodologies were utilized to identify optimal feature between ligands.

In the process of pharmacophore production, multi-objective function in which each term (SE, SO, and PhS) was considered independently. The multi-objective functions were employed to assess ability of producing pharmacophore characteristics, and these functions were also beneficial to select candidates that survived to the next generation and to rank models after Cartesian alignment of compounds conformations. Finally, 20 models were generated with different features.

Intrinsic parameters were calculated to evaluate the generated pharmacophore models. Each pharmacophore model has seven parameters: Specificity, N_hits, Pareto, Energy, Sterics, Hbond, and Mol_qry. Specificity is an index for the expected discrimination of each model, based on the number of features it contained. The number of “hits” (N_hits) represents the number of compounds in the training set that the model hits. Pareto value indicates the Pareto rank of each model. A value of zero indicates there is no model superior to any others. Energy and Sterics represent the total energy and the steric overlap of the model. Hydrogen Bonds (Hbond) indicates the pharmacophoric concordance. Molecular query (Mol_qry) quantifies the agreement between the query tuple and pharmacophore. Due to the conflict between above intrinsic parameters of the obtained models, ∑Ranking was used to evaluate all indicators synthetically, and the model which possesses the lowest ∑Ranking value was regarded as the optimal model [19]. 

### 3.2. Homology Modeling Studies

Homology modeling process is divided into three steps: (1) The amino acid sequences of mGluRs were downloaded from the UniProt database (http://www.uniprot.org/), and the sequences were used as the query to screen possible template proteins from the Protein Data Bank (http://www.rcsb.org/) by BLAST tool from NCBI (https://blast.ncbi.nlm.nih.gov/blast.cgi), respectively. (2) The template proteins were utilized as templates to build models by MODELLER program. The Looper algorithm was used to refine the loop domains of the models. (3) The refined models were obtained.

The refined models were further optimized by molecular dynamics (MD) simulation. The refined models were selected as the start pose of MD with water environment. The MD simulations were run with GROMACS 5.0.2 using the GROMOS96 43a1 force field [41]. The system was subjected to the CHARMm force-field and relaxed by energy minimization using 5000 steps of steepest descent integrator. Next, A 500 ps NVT equilibration was performed at temperature of 300 K with position restraints applied to protein in order to relieve any bad contacts at the residues solvent interface [42]. Then a 1000 ps NPT simulation was conducted, and pressure was coupled to 1.0 atm using the Parrinello–Rahman method. After completing the two equilibrium phases, the position constraints were released and the MD was subjected to 15 ns using the v-recalibration method and the Parrinello–Rahman method. The snapshot structures and energies were saved for every 10 ps. The conformation with the lowest energy was selected as the optimal model for each mGluRs and then performed the energy minimization.

Ramachandran plot and ERRAT were used to assess the models. The Ramachandran plot is often used as the first check to verify predicted torsion angles of protein models by analyzing residue-by-residue geometry and overall structure geometry. The presence of less than 20% of the residues in the unfavorable regions indicates that the structures are reasonable [43]. ERRAT was calculated to further evaluate the reliability of the models by analyzing the non-bonded interactions between different atom types and comparing with highly refined structures [21].

### 3.3. Molecular Docking

Surflex-Dock was used to analyze the interaction between ligands and proteins [42]. As a commonly used molecular docking method, it has the characteristics of docking accuracy and screening utility. Because of the specificity of the crystal structures of each subtype, the proteins need to be prepared differently. Different methods were also utilized to define the active pocket in SYBYL-X2.1.1. 

A Protomol used to guide docking of the molecules is a computational representation of the expected binding site to which the putative ligand is aligned [44]. The protomols was generated by one of three routes [45]: (1) automatic: Surflex-Dock are able to find the largest cavity in the receptor protein; (2) ligand-based: a small molecule that fits into the site of interest are selected to use to generate protomol; (3) residue-based: specified residues in the receptor are selected to generate protomol. The two important factors that can significantly affect the size and extent of the protomol generated are Threshold and Bloat. Threshold determines how far the protomol extends into the concavity of the target site, while Bloat impacts how far the protomol extends outside of the concavity.

For mGluRs family, the crystal structures of extracellular domains (orthosteric binding site) of five subtypes have been reported, including mGluR1 (PDB ID: 3KS9; resolution, 1.9 Å), mGluR5 (PDB ID: 3LMK; resolution, 2.44 Å), mGluR2 (PDB ID: 4XAQ; resolution, 2.21 Å), mGluR3 (PDB ID: 4XAR; resolution, 2.26 Å), and mGluR7 (PDB ID: 3MQ4; resolution, 2.8 Å). However, only mGluR1 and mGluR5 have the crystal structures of 7TMD contains the allosteric binding site, including mGluR1 (PDB ID: 4OR2; resolution, 2.8 Å) and mGluR5 (PDB ID: 4OO9; resolution, 2.6 Å). Those crystal structures were downloaded from the PDB, common preparation was automatically solved, such as adding hydrogen, removing water, extra protein chains, and extracting the initial ligands [46]. The initial ligands extracted from the crystal structures were used to define active pockets. 

For the homology modeling models of extracellular domains of mGluR4 and mGluR8, which built based on the same template protein 3MQ4, the initial ligand (Z99) of the template protein was retained. Z99 has been discovered as antagonist of mGluR ІІІ, and has antagonist activity for mGluR4 and mGluR8. Therefore, the initial ligand (Z99) was used to define active pockets of mGluR4 and mGluR8. The binding sites of the others homologous modeling structures were defined with amino acids confirmed by literatures [27] with the radius set to 5 Å. The amino acids of the proteins are shown in Table S12. The detailed parameters of protomol are shown in the Table S13.

### 3.4. Data Analysis

The TCMs of potential orthosteric or allosteric compounds of mGluRs were traced. In this process, the duplicated TCMs of each group will be deleted. Then, the five flavors of the remained TCMs were analyzed, and the highly frequency flavors were found out. Besides this, the pharmacological effect of TCMs which corresponding the highly frequency flavors were collected, and the representative effects were summed up and analyzed. Finally, the comprehensive analysis of correlation of target–five flavors–functions was performed to explain the relationship between flavors and neuroprotection of TCMs based on the mGluRs.

## 4. Conclusions

According to the traditional Chinese medicine theory, the traditional pharmacological effect of TCMs is closely related to the five flavors. However, the relationship between modern pharmacological effect on TCMs and its five flavors is still not clear. In this paper, the relationship between mechanism of neuroprotection and five flavors of TCMs was discussed based on mGluRs. Molecular simulation models of the mGluRs were built, including six pharmacophore models of three groups based on their orthosteric and allosteric site, seven homology modeling models, and fourteen molecular docking models. 

Based on virtual screening, potential compounds which bound to orthosteric site and allosteric site of three groups of mGluRs were obtained respectively. Then, the original TCMs of potential compounds were traced. Interestingly, the TCMs of compounds that act on the orthosteric site of the mGluRs usually mainly have the property of sweet flavor, while the compounds bound to allosteric site of the mGluRs mostly come from TCMs with bitter flavor. The functions of the highly frequency TCMs were divided into eight classes, including analgesic effect (Zhi Tong in Chinese Pinyin), clearing toxic materials (Jie Du), dispelling dampness (Qu Shi), detumescence (Xiao Zhong), promoting blood circulation (Huo Xue), dispelling wind (Qu Feng), and eliminating cold (San Han). Thus, TCMs acting on orthosteric and allosteric sites of mGluRs have different flavors and traditional pharmacological effects, but these TCMs could also produce the similar neuroprotective effect. 

This paper provides the scientific support for the developing new drugs of neuroprotective TCMs which target mGluRs. Besides this, it is possible to explain neuroprotective mechanism of TCM through the mGluRs. The methods that distinguish the flavors of TCM through the orthosteric and allosteric ligands of taste receptors, provide a novel approach to explore the flavors of TCM. Besides this, the mechanism of Jieyu Hehuan decoction was speculated based on the result of molecular simulation.

## Figures and Tables

**Table 1:** The result of the pharmacophore models of orthosteric antagonist of mGluR I.

Model	Specificity	N_HITS	PARETP	Energy	Sterics	HBOND	MOL-QRY
model 01	3.458	6	0	10.79	203.10	107.90	2.29
model 02	3.456	6	0	4.52	193.60	101.10	2.90
**model 03 ^a^**	**3.458**	**6**	**0**	**13.70**	**203.10**	**107.90**	**2.29**
model 11	3.458	6	0	14.14	200.50	108.90	2.29
model 13	3.454	6	0	16.61	203.30	107.40	2.15
model 15	3.451	6	0	3.73	187.10	107.70	0.66
model 16	3.452	6	0	6.98	188.50	102.70	2.29
model 17	3.452	6	0	8.95	195.10	99.40	2.29
model 18	3.452	6	0	10.72	187.40	104.70	2.29

^a^ Bold text refer to the optimal model.

**Table 2:** The ranking results of orthosteric GALAHAD models of mGluR I.

Model	Specificity	Energy	Sterics	HBOND	MOL-QRY	∑Ranking
model 01	1	7	2	2	2	15
model 02	4	2	6	8	1	22
**model 03 ^a^**	**1**	**6**	**2**	**2**	**2**	**14**
model 11	1	8	4	1	2	17
model 13	4	9	1	5	6	26
model 15	4	1	9	4	7	26
model 16	7	3	7	7	2	27
model 17	7	4	5	9	2	28
model 18	7	5	8	6	2	29

^a^ Bold text refer to the optimal model.

**Table 3:** The results of database search.

	Number of Compounds	Pharmacophore Screening	Drug-Like Compounds	Blood–Brain Barrier Permeability
Crystal Structure				
mGluR I	Orthoteric site	123	89	43
	Allosteric site	320	221	94
mGluR II	Orthoteric site	178	103	30
	Allosteric site	532	287	105
mGluR III	Orthoteric site	110	80	3
	Allosteric site	1714	392	23

**Table 4:** Selection of template proteins and evaluation of models.

Domain	Target	Templates	Identity Value	Ramachandran Plot	ERRAT
Extracellular Domain	mGluR4	3MQ4	99%	93.79%	80.000
	mGluR8	3MQ4	72%	94.26%	83.559
7TMD	mGluR2	4OR2	52%	99.60%	89.879
	mGluR3	4OO9	47%	95.70%	87.391
	mGluR4	4OR2	43%	98.43%	94.400
	mGluR7	4OO9	43%	98.11%	89.453
	mGluR8	5CGC	44%	100%	88.462

**Table 5:** The data of correlation between the rank of compounds experimental affinity (Ki) and docking score of mGluR4.

No_Compounds	Ki	Rank of Ki	Total Score	Rank of Total Score
CHEMBL33567	910	1	7.2647	2
CHEMBL277475	5200	2	7.4445	1
CHEMBL329236	8800	3	6.6985	3
CHEMBL89000	21,000	4	6.6781	4
CHEMBL90501	23,000	5	6.1632	5
CHEMBL88612	26,000	6	4.3929	7
CHEMBL41221	470,000	7	5.6421	6
Correlation	0.9286			

**Table 6:** The data of correlation between the rank of compounds experimental affinity (Ki) and docking score of mGluR7.

No_Compounds	Ki (nM)	Rank of Ki	Total Score	Rank of Total Score
CHEMBL33567	175,000	1	6.8675	1
CHEMBL277475	185,000	2	6.4943	2
BDBM17657	5,400,000	3	5.215	3
Correlation	1.0000			

**Table 7:** The data of correlation between the rank of compounds experimental affinity (Ki) and docking score of mGluR8.

No_Compounds	Ki	Rank of Ki	Total Score	Rank of Total Score
CHEMBL33567	61	1	6.3650	2
CHEMBL277475	210	2	6.9249	1
CHEMBL89000	1700	3	6.0272	3
CHEMBL280563	3400	4	5.9511	4
CHEMBL88999	7300	5	5.1538	9
BDBM17657	9500	6	4.6927	5
CHEMBL8759	12,000	7	4.1048	6
CHEMBL330097	15,000	8	3.6829	7
CHEMBL34453	45,000	9	5.9186	8
Correlation	0.8167			

**Table 8:** Identification and evaluation of the orthosteric active pockets.

Domian	Target	Crystal Structure	Initial Ligand	RMSD/Correlation ^a^
Extracellular Domain	mGluR1	3KS9	Z99	1.4946 Å
	mGluR5	3LMK	NAG	/
	mGluR2	4XAQ	40F	1.1403 Å
	mGluR3	4XAR	40F	0.5063 Å
	mGluR4	Homology Model	Z99	0.9286
	MGluR7	3MQ4		1.0000
	MGluR8	Homology Model		0.8167

^a^ Plain and bold text refer to RMSD and Correlation.

**Table 9:** The identification and rationality of the allosteric active pocket.

Domian	Target	Crystal Structure	Define Pocket	RMSD
7TMD	mGluR1	4OR2	FM9	1.6314 Å
	mGluR5	4OO9	2U8	1.0754 Å
	mGluR2	Homology Model	amino acids	/
	mGluR3	Homology Model	amino acids	/
	mGluR4	Homology Model	amino acids	/
	mGluR7	Homology Model	amino acids	/
	mGluR8	Homology Model	amino acids	/

## Supplementary Materials

Supplementary materials can be found at www.mdpi.com/1422-0067/19/1/163/s1.
