# Supplementary material for: In Silico Analysis of the Association Relationship between Neuroprotection and Flavors of Traditional Chinese Medicine Based on the mGluRs
Source: Int J Mol Sci. 2018 Jan 10;19(1):163. doi: 10.3390/ijms19010163 (PMC5796112; doi:10.3390/ijms19010163)
Supplement: Supplementary file 1 [file ijms-19-00163-s001.pdf]

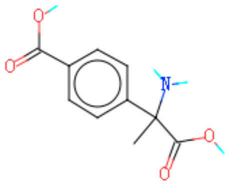

Compound 1[a]

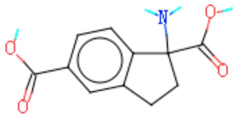

Compound 2[a]

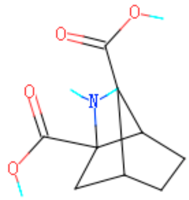

Compound 3[a]

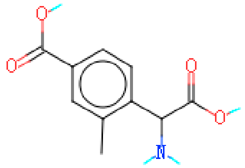

Compound 4[a]

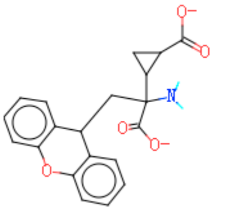

Compound 5[a]

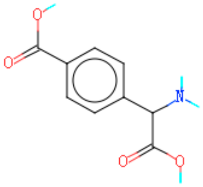

Compound 6[a]

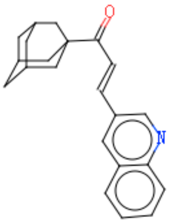

Compound 7[b]

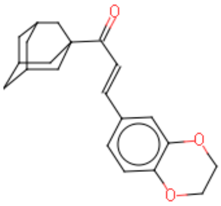

Compound 8[b]

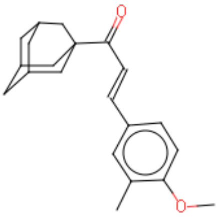

Compound 9[b]

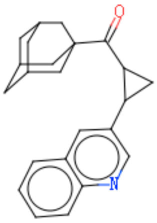

Compound 10[b]

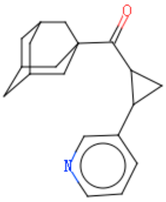

Compound 11[b]

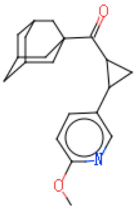

Compound 12[b]

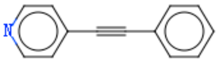

Compound 13[b]

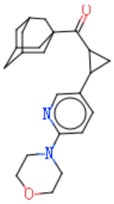

Compound 14[b]

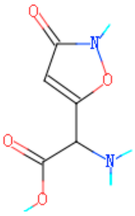

Compound 15[c]

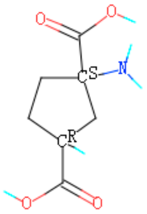

Compound 16[c]

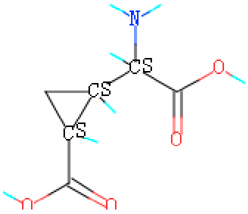

Compound 17[c]

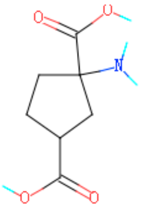

Compound 18[c]

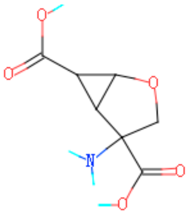

Compound 19[c]

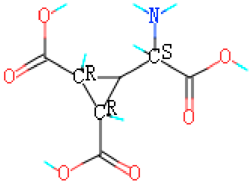

Compound 20[c]

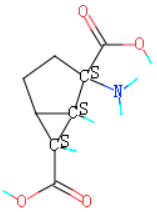

Compound 21[c]

3

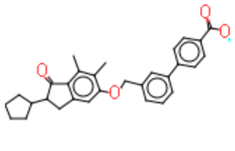

Compound 22[d]

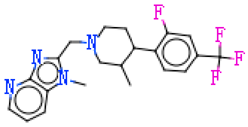

Compound 23[d]

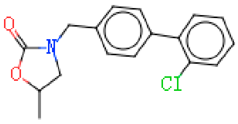

Compound 24[d]

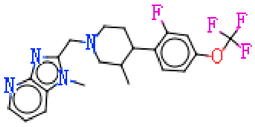

Compound 25[d]

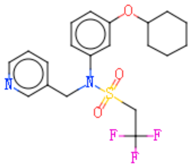

Compound 26[d]

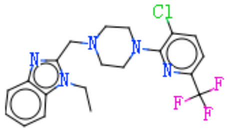

Compound 27[d]

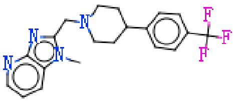

Compound 28[d]

4

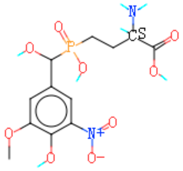

Compound 29[e]

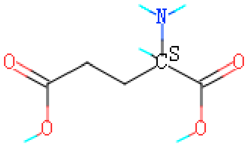

Compound 30[e]

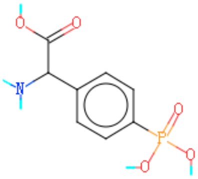

Compound 31[e]

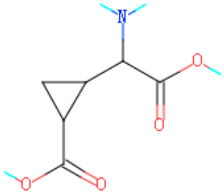

Compound 32[e]

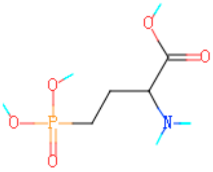

Compound 33[e]

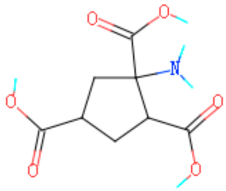

Compound 34[e]

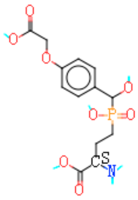

Compound 35[e]

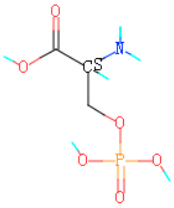

Compound 36[e]

5

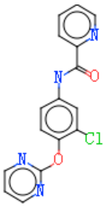

Compound 37[f]

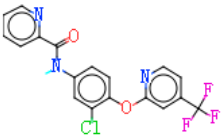

Compound 38[f]

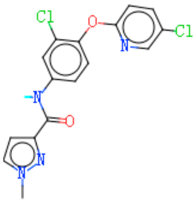

Compound 39[f]

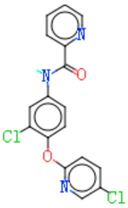

Compound 40[f]

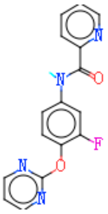

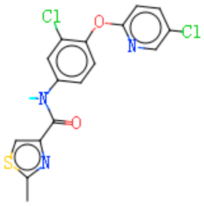

Compound 41[f] Compound 42[f]

Figure S1. Structures of orthosteric and allosteric compounds which used to generate pharmacophore of mGluRs.(a) Structures of 6 antagonists which used to generate orthosteric pharmacophore of mGluRⅠ; (b) Structures of 8 NAMs which used to generate allosteric pharmacophore of mGluRⅠ;(c) Structures of 7 agonists which used to generate orthosteric pharmacophore of mGluRⅡ;(d) Structures of 7 PAMs which used to generate allosteric pharmacophore of mGluRⅡ; (e) Structures of 7 agonists which used to generate orthosteric pharmacophore of mGluRIII; (f) Structures of 6 PAMS which used to generate allosteric pharmacophore of mGluRIII.

Table S1. The results of orthosteric GALAHAD models of mGluRⅡ.

Model	Specificity	N_HITS	PARETP	Energy	Sterics	HBOND	MOL-QRY
**Model 06[a]**	**3.837**	**6**	**0**	**9.08**	**67.20**	**84.80**	**2.37**
Model 09	3.398	6	0	8.10	65.90	84.50	3.11
model 10	2.863	7	0	13.74	66.20	85.10	0.35
model 20	3.993	6	0	4.12	59.50	82.40	1.58

[a] Bold text refer to the optimal model.

Table S2. The results of allosteric GALAHAD models of mGluRⅡ.

Model	Specificity	N_HITS	PARETP	Energy	Sterics	HBOND	MOL-QRY
model 03	2.753	7	0	38.21	384.9	100	14.8
**model 09 [a]**	**4.106**	**7**	**0**	**22.59**	**375.9**	**98.9**	**6.5**

[a] Bold text refer to the optimal model.

Table S3. The results of orthosteric GALAHAD models of mGluRIII.

Model	Specificity	N_HITS	Pareto	Energy	Sterics	Hbond	MOL-QRY
model 01	3.109	8	0	8.62	188	199.1	12.07
model 08	3.020	8	0	6.35	175	188.2	4.28
model 10	3.010	8	0	2621.4199	212.8	188.9	2.29
model 11	4.049	8	0	7.86	188	162.3	21.8
model 13	3.006	8	0	5.45	182.5	170.4	3.52
model 14	2.603	8	0	2.34	160.8	162.7	10.15
**model 17 [a]**	**4.065**	**8**	**0**	**8.60**	**207.6**	**163.5**	**4.63**

[a] Bold text refer to the optimal model.

Table S4. The results of allthosteric GALAHAD models of mGluRIII.

Model	Specificity	N_HITS	PARETP	Energy	Sterics	HBOND	MOL-QRY
model 01	3.248	6	0	5.56	653.60	237.40	218.63
**model 04 [a]**	**4.666**	**6**	**0**	**4.08**	**587.90**	**229.60**	**173.15**

model 05	4.415	6	0	5.09	533.90	243.50	182.88
model 08	3.397	6	0	4.52	583.30	239.90	141.91
model 09	4.426	6	0	3.67	569.20	208.50	160.27
model 10	4.400	6	0	6.38	670.00	229.80	140.23
model 19	3.462	6	0	3.32	479.90	205.50	130.44

[a] Bold text refer to the optimal model.

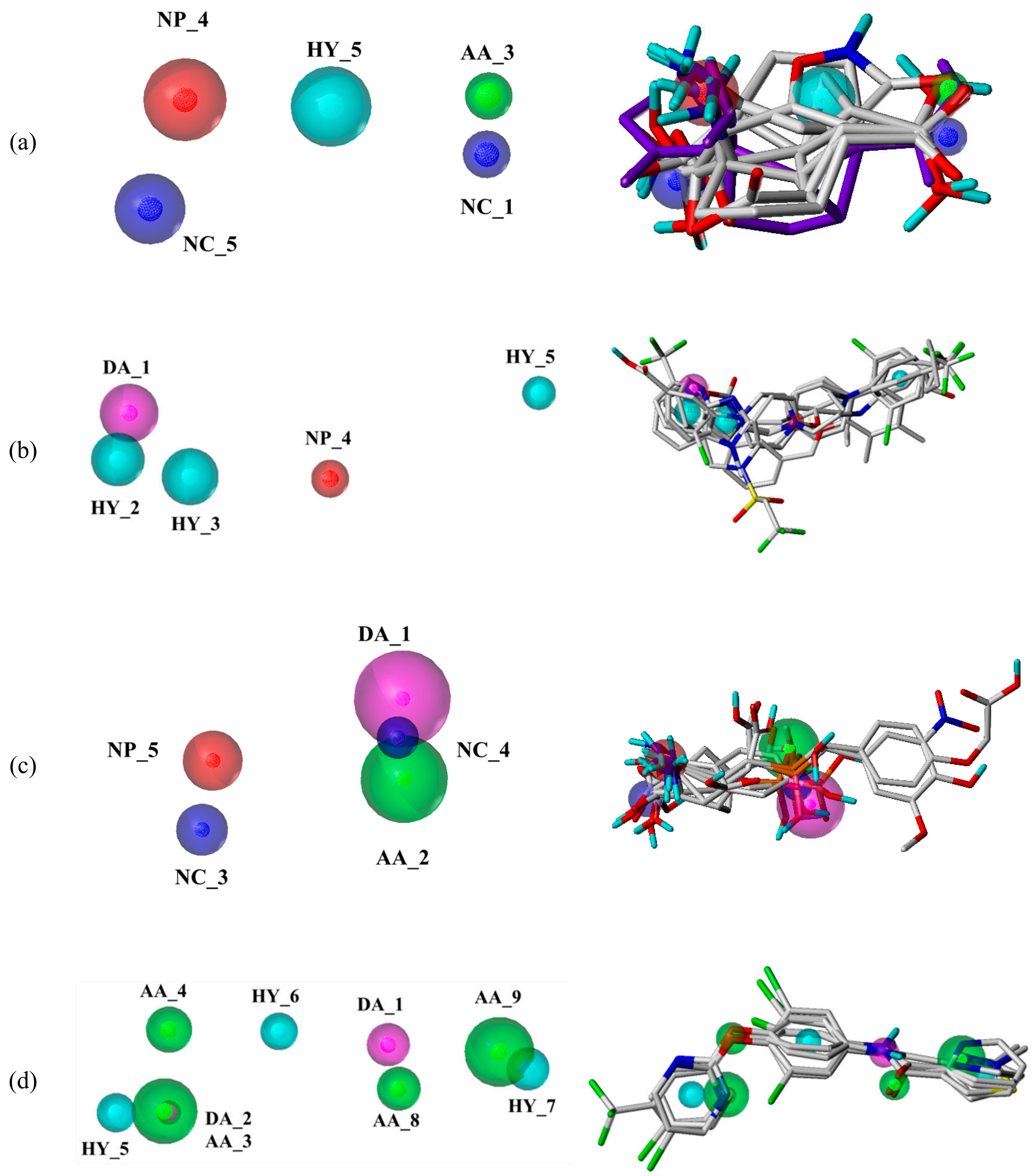

Figure S2. The optimal pharmacophore model of mGluRs and mapped with the compounds which used to build model. (a)The optimal pharmacophore model of orthosteric site of mGluRⅡ; (b)The optimal pharmacophore model of allosteric site of mGluRⅡ; (C)The optimal pharmacophore model of orthosteric site of mGluRⅢ; (d)The optimal pharmacophore model of allosteric site of mGluRⅢ.

(a)

(b)

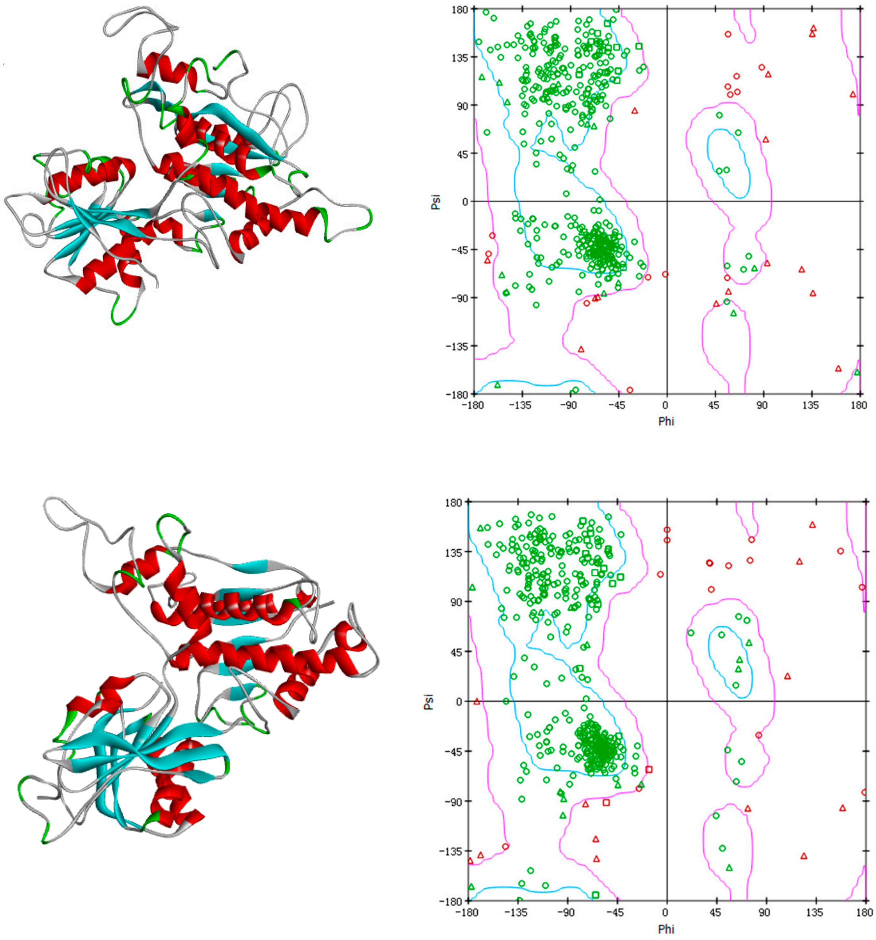

Figure S3. The crystal structures of the extracellular domain of mGluRs and the ramachandran plot results of them. (a)mGluR4; (b) mGluR8

(a)

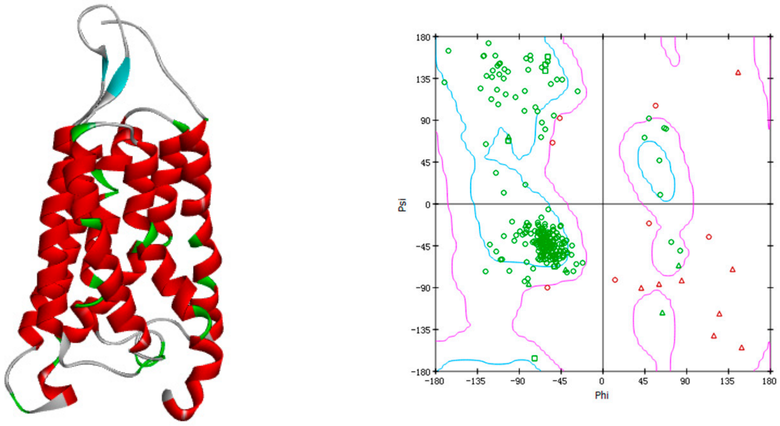

(b)

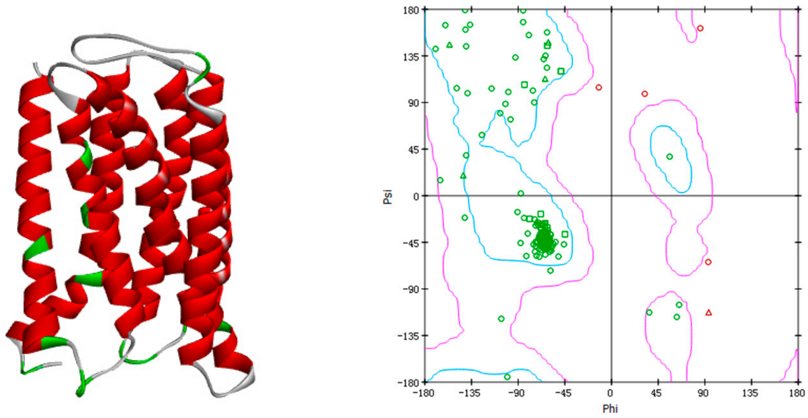
180
135
90
45
0
−45
−90
−135
−180
Psi
−180 −135 −90 −45 0 45 90 135 180
Phi

(c)

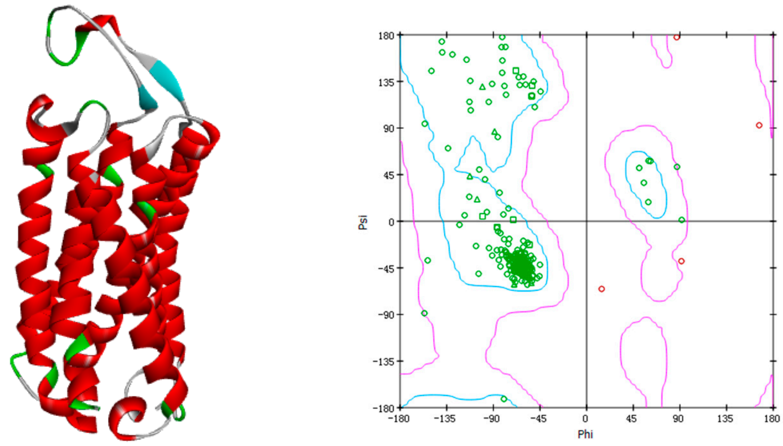
180
135
90
45
0
−45
−90
−135
−180
Psi
−180 −135 −90 −45 0 45 90 135 180
Phi

(d)

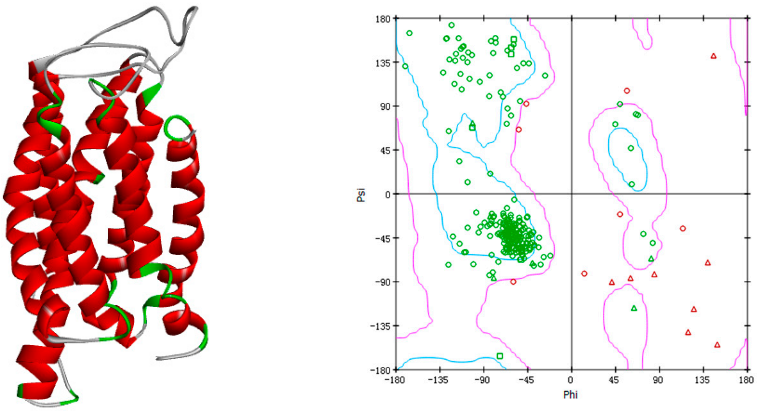
180
135
90
45
0
−45
−90
−135
−180
Psi
−180 −135 −90 −45 0 45 90 135 180
Phi

(e)

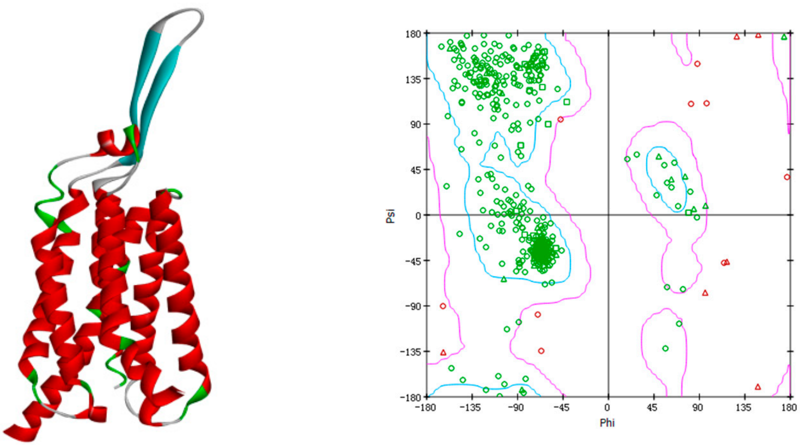
180
135
90
45
0
−45
−90
−135
−180
Psi
−180 −135 −90 −45 0 45 90 135 180
Phi

Figure S4. The crystal structures of the 7TMD domain of mGluRs and the ramachandran plot results of them. (a) mGluR2; (b) mGluR3 ;(c)mGluR4; (b) mGluR7; (e) mGluR8

Table S5. The flavors results of source TCMs corresponding orthosteric site of mGluRs Ⅰ

Latin Name (Chinses pinyin name)	sour	bitter	sweet	pungent	salty
Abrus cantoniensis Hance(Ji Gu Cao)	0	1	1	0	0
A.Sativam L. var．Viviparum Regel(Da Suan)	0	0	0	1	0
Mylabris(Ban Mao)	0	0	0	1	0
Semen Trigonellae(Hu Lu Ba)	0	1	0	0	0
Fructus Mori(Sang Shen)	0	0	1	0	1
Juncus effusus L.(Deng Xin Cao)	0	0	1	0	0
Radix Aucklandiae(Mu Xiang)	0	1	0	1	0
Folium Isatidis(Da Qing Ye)	0	1	0	0	0
Fructus Lycii(Gou Qi Zi)	0	0	1	0	0
Caulis Piperis Kadsurae(Hai Feng Teng)	0	1	0	1	0
Reynoutria japonica Houtt.(Hu Zhang)	0	1	0	0	0
Atractylodes lancea (Thunb.) DC.(Cang Zhu)	0	1	0	1	0
Cortex Albizziae(He Huan Pi)	0	0	1	0	0
Veneum Bufonis(Chan Su)	0	0	0	1	0
Zingiber officinale Rosc.(Sheng Jiang)	0	0	0	1	0
Semen Gingko(Bai Guo)	0	1	1	0	0
Dolichos lablab L.(Bai Bian Dou)	0	0	1	0	0
Litchi chinensis Sonn(Li Zhi He)	0	1	1	0	0
Codonopsis pilosula (Franch.) Nannf.(Dang Shen)	0	0	1	0	0
**Total**	0	9	9	7	1

Table S6. The flavors results of source TCMs corresponding orthosteric site of mGluR Ⅱ

Latin Name (Chinses pinyin name)	sour	bitter	sweet	pungent	salty
Mylabris(Ban Mao)	0	0	0	1	0
Areca catechu Linn.(Bing Lang)	0	1	0	1	0
Angelica sinensis (Oliv.) Diels(Dang Gui)	0	0	1	1	0
Codonopsis pilosula (Franch.) Nannf.(Dang Shen)	0	0	1	0	0
Cortex Albizziae(He Huan Pi)	0	0	1	0	0
Semen Ricini Ricinus communis L.(Bi Ma Zi)	0	0	1	1	0
Veneum Bufonis(Chan Su)	0	0	0	1	0
Uncaria rhynchophylla (Miq.) Miq. ex Havil.(Gou Teng)	0	0	1	0	0
Fructus Mori(Sang Shen)	0	0	1	0	1
Folium Artemisiae Argy(Ai Ye)	0	1	0	1	0
**Total**	0	2	6	6	1

Table S7. The flavors results of source TCMs corresponding orthosteric site of mGluRⅢ

Latin Name (Chinses pinyin name)	sour	bitter	sweet	pungent	salty

Cortex Albizziae(He Huan Pi)	0	0	1	0	0
Portulaca oleracea Linn.(Ma Chi Xian)	1	0	0	0	0
Semen Trigonellae(Hu Lu Ba)	0	1	0	0	0
Apis cerana(Feng Mi)	0	0	1	0	0
**Total**	1	1	2	0	0

Table S8. The flavors results of source TCMs corresponding allosteric site of mGluRs Ⅰ

Latin Name (Chinses pinyin name)	sour	bitter	sweet	pungent	salty
Glycine max (L.) Merr.(Hei Dou)	0	0	1	0	0
Glycyrrhiza uralensis Fisch.(Gan Cao)	0	0	1	0	0
Semen Trigonellae(Hu Lu Ba)	0	1	0	0	0
Paeonia lactiflora (Bai Zhi)	0	0	0	1	0
Cortex Meliae(Ku Lian Pi)	0	1	0	0	0
Radix Paeoniae Alba(Bai Shao)	1	1	0	0	0
Psoralea corylifolia Linn.(Bu Gu Zhi)	0	1	0	1	0
Uncaria rhynchophylla (Miq.) Miq. ex Havil.(Gou Teng)	0	0	1	0	0
Artemisia capillaris (Yin Chen)	0	1	0	1	0
Fritillaria thunbergii(Zhe Bei Mu)	0	1	0	0	0
Magnolia liliiflora Desr(Xin Yi)	0	0	0	1	0
Notopterygium incisum(Qiang Huo)	0	0	0	1	0
Vitex trifolia L. (Man Jing Zi)	0	1	0	1	0
Uriospora capillipes Gagnep(Jin Guo Lan)	0	1	0	0	0
**Total**	1	9	3	9	0

Table S9. The flavors results of source TCMs corresponding allosteric site of mGluR Ⅱ

Latin Name (Chinses pinyin name)	sour	bitter	sweet	pungent	salty
Corydalis decumbens (Thunb.) Pers.(Xia Tian Wu)	0	1	0	1	0
Aconitum carmichaelii Debx.(Fu Zi)	0	0	1	1	0
Ferula sinkiangensis K. M. Shen (A WEI)	0	1	0	1	0
Hyoscyamus niger L.(Tian Xian Zi)	0	1	0	1	0
Atropa belladonna L.(Dian Qie Cao)	0	1	0	1	0
Corydalis bungeana Turcz(Ku Di Ding)	0	1	0	0	0
Bfoussonetia papyri fera (L.)Vent (Chu Shi Zi)	0	0	1	0	0
Psoralea corylifolia Linn.(Bu Gu Zhi)	0	1	0	1	0
Aconitum carmichaelii Debx.(Chuan Wu)	0	1	0	1	0
Aconitum kusnezoffii Reichb.(Cao Wu)	0	1	0	1	0
Vigna angularis (Willd.) Ohwi et Ohashi(Chi Xiao Dou)	1	0	1	0	0
Murraya exotica L. Mant.(Jiu Li Xiang)	0	1	0	1	0

vodia rutaecarpa (Juss.) Benth.(Wu Zhu Yu)	0	1	0	1	0
Allium macrostemon Bunge(Xie Bai)	0	1	0	1	0
Corrydalis yanhusuo W. T.W a n g(Yan Hu Suo)	0	1	0	1	0
Corydalis decumbens (Thunb.) Pers.(Xia Tian Wu)	0	1	0	1	0
Glycyrrhiza uralensis Fisch.(Gan Cao)	0	0	1	0	0
Picrasma quassioides (D. Don)Benn.(Ku Mu)	0	1	0	0	0
**Total**	1	13	4	12	0

Table S10. The flavors results of source TCMs corresponding allosteric site of mGluRIII

Latin Name (Chinses pinyin name)	sour	bitter	sweet	pungent	salty
Zingiber officinale Rosc.(Sheng Jiang)	0	0	0	1	0
Murraya exotica L. Mant.(Jiu Li Xiang ）	0	1	0	1	0
Picrasma quassioides(D． Don)Benn． (Ku Mu)	0	1	0	0	0
Allium macrostemon Bunge(Xie Bai)	0	1	0	1	0
Paeonia lactiflora (Bai Zhi)	0	0	0	1	0
Cortex Meliae(Ku Lian Pi)	0	1	0	0	0
**Total**	0	4	0	4	0

Table S11. The top 10 most frequent efficiencies.

	mGluR I		mGluR II		mGluR III	
Orthosteric	Zhi Tong	5	Zhi Tong	3	Jie Du	1
	Jie Du	3	Xiao Zhong	2	Zhi Tong	1
	Qu Shi	3	Huo Xue	2	Huo Xue	1
	Qing Re	3	Gong Du	2	Xiao Zhong	1
	Jian Pi	3	Jie Du	1	Jie Yu	1
	San Jie	2	Kai Qiao	1	An Shen	1
	Zhi Ke	2	Xi Feng	1	Lian Chuang	1
	Zhu Yang	2	Zhi Xue	1	Sheng Ji	1
	Bu Shen	2	Hua Yu	1		
	Xing Qi	2	Ba Du	1		
Allosteric	Zhi Tong	7	Zhi Tong	7	Qu Shi	2
	Qu Feng	4	Qu Shi	4	Zhi Tong	2
	San Han	4	Qu Feng	3	San Han	2
	Qing Re	2	Xing Qi	3	Hua Tan	2
	Qu Shi	2	Huo Xue	3	Jie Biao	2
	Xiao Zhong	2	Jie Du	2	Xing Qi	2
	Jie Du	2	Qing Re	2	Jie Du	1
	Wen Shen	2	Zhu Yang	2	Huo Xue	1
	Jie Biao	2	Ping Chuan	2	Qu Feng	1
	Xiao Ji	2	San Jie	2	Qing Re	1

Table S12 The amino acids used to define the active pockets.

mGluR2	mGluR3	mGluR4	mGluR7	mGluR8
Gly612	Gly621	Gly632	Gly635	Gly628
Val613	Val622	Ile633	Ile636	Ile629
Cys616	Ser625	Cys636	Cys639	Cys632
Phe623	Phe632	Met643	Met646	Met639
Arg635	Arg644	Arg655	Arg658	Arg651
Leu639	Leu648	Leu659	Leu662	Leu655
Phe643	Phe652	Met663	Met666	Met659
Try647	Try656	Try667	Try670	Try663
Met728	Met737	Leu753	Ile756	Leu749
Ser731	Ser740	Leu756	Ser759	Ser752
Leu732	Leu741	Leu757	Leu760	Leu753
Asn735	Asp744	Ser760	Ser763	Ser756
Ile739	Val748	Met764	Met767	Met760
Thr769	Thr778	Thr794	Thr797	Thr790
Ile772	Ile781	Val797	Val800	Ile793
Trp773	Trp782	Trp798	Trp801	Trp794
Phe776	Phe785	Phe801	Phe804	Phe797
Phe780	Phe789	Phe805	Phe808	Phe801
Met794	Met803	Leu822	Leu825	Leu818
Ser797	Ser806	Ser825	Ser828	Ser821
Val798	Val807	Val826	Met829	Met822
Ser801	Ser810	Ser829	Ser832	Ser825

Table S13 The detailed parameters of protomol.

Domian	Target	Threshold	Bloat
Extracellular Domain	mGluR1	0.6	0
	mGluR2	0.5	0
	mGluR3	0.5	0
	mGluR4	0.5	0
	mGluR5	0.5	0
	mGluR7	0.5	0
	mGluR8	0.5	0
7TMD	mGluR1	0.6	0
	mGluR2	0.5	0
	mGluR3	0.5	0
	mGluR4	0.5	0

mGluR5	0.4	0
mGluR7	0.5	0
mGluR8	0.5	0

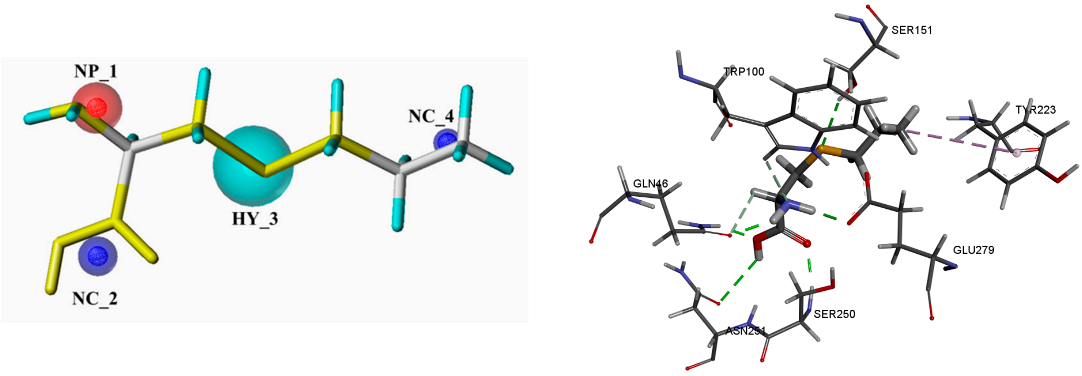

Figure S5. Pharmacophore mapping results and molecular docking results of S-Propyl-L-cystein (orthosteric site of mGluR5)

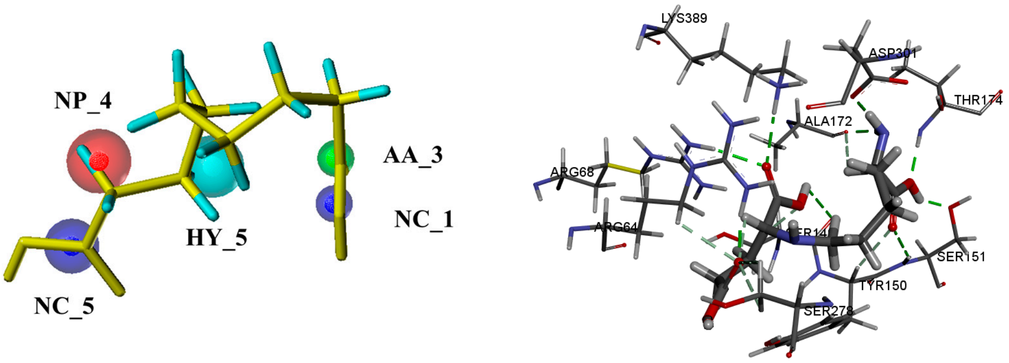

Figure S6. Pharmacophore mapping results and molecular docking results of Azedarachin C (orthosteric site of mGluR2)

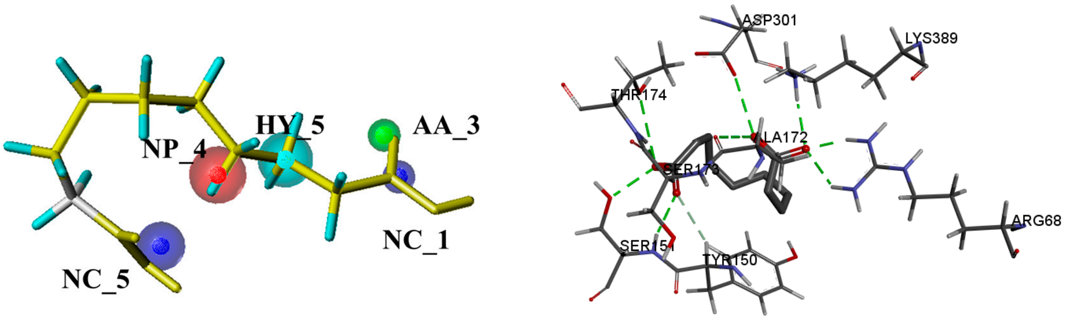

Figure S7. Pharmacophore mapping results and molecular docking results of D-Rhodoic acid (orthosteric site of mGluR3)

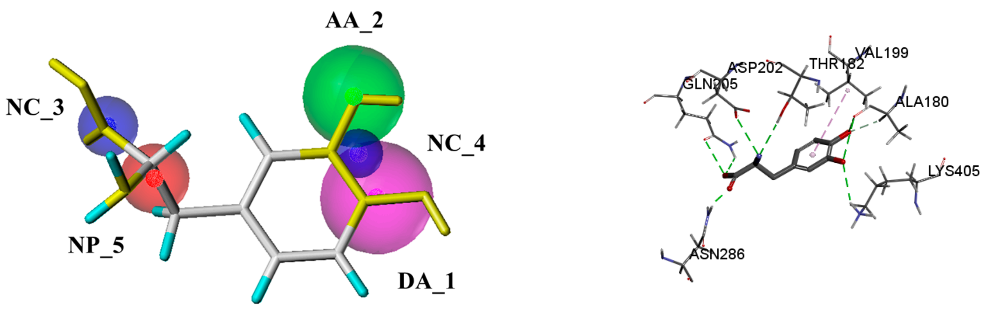

Figure S8. Pharmacophore mapping results and molecular docking results of Dopa (orthosteric site of mGluR4)

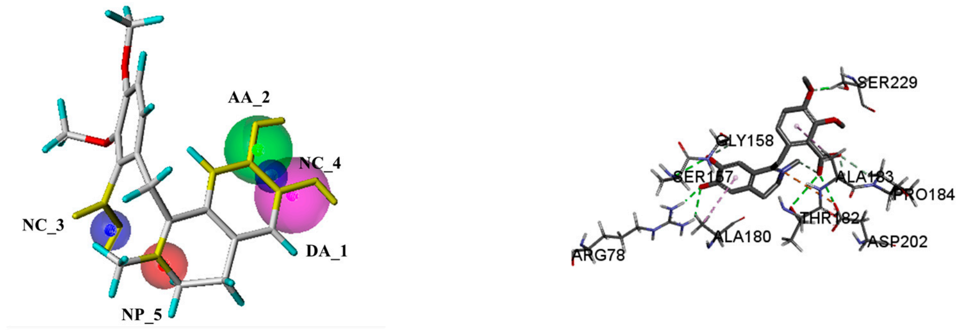

Figure S9. Pharmacophore mapping results and molecular docking results of Leptopinine (orthosteric site of mGluR7)

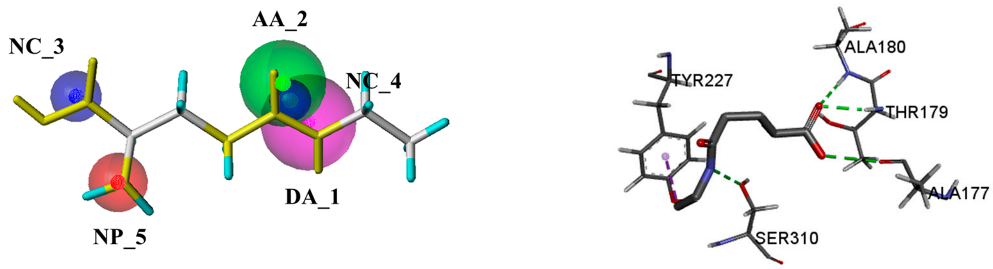

Figure S10. Pharmacophore mapping results and molecular docking results of Theanine (orthosteric site of mGluR8)

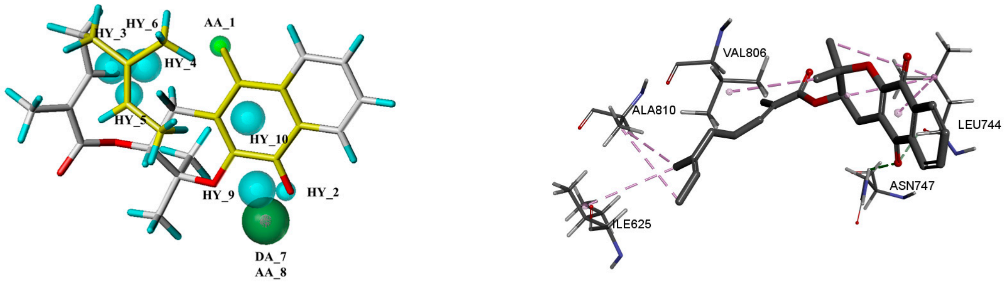

Figure S11. Pharmacophore mapping results and molecular docking results of Rhinacanthin B (allosteric site of mGluR5)

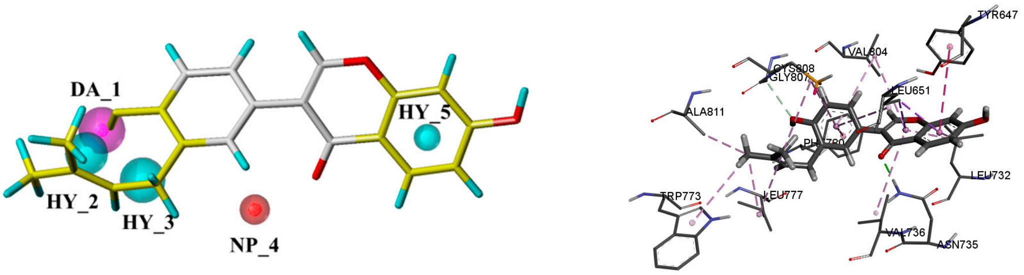

Figure S12. Pharmacophore mapping results and molecular docking results of Neobavaisoflavone (allosteric site of mGluR2)

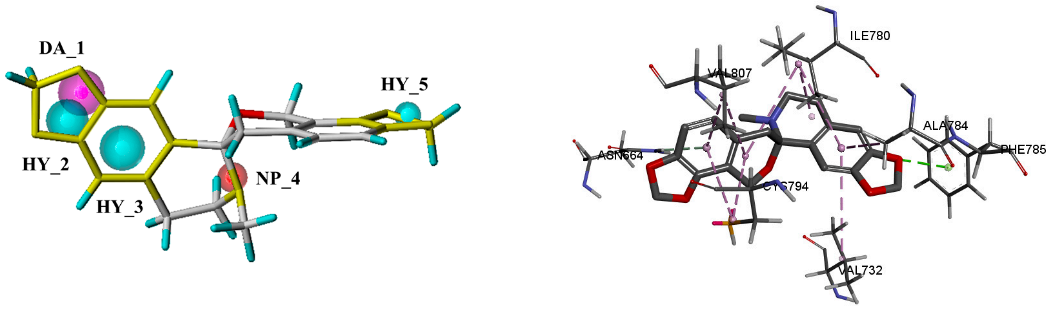

Figure S13. Pharmacophore mapping results and molecular docking results of Hypecorine (allosteric site of mGluR3)

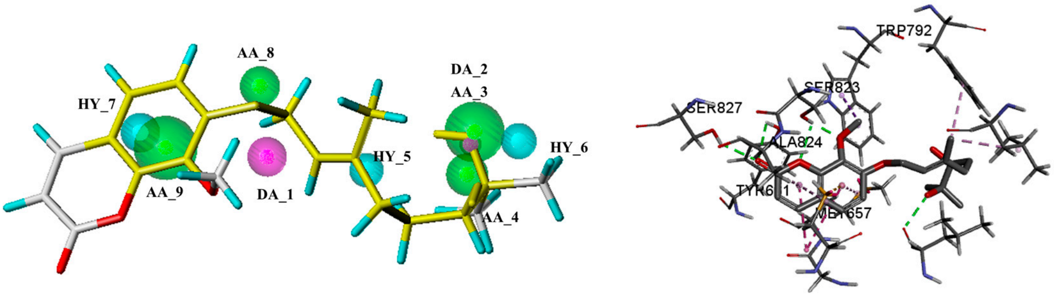

Figure S14. Pharmacophore mapping results and molecular docking results of Schinindiol (allthosteric site of mGluR4)

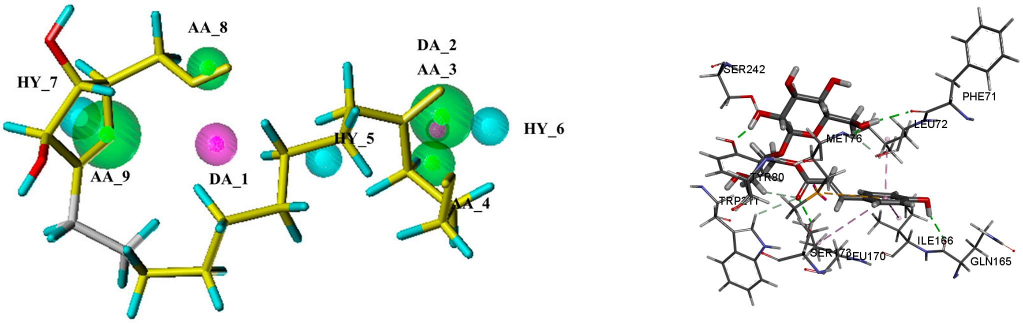

Figure S15. Pharmacophore mapping results and molecular docking results of Broussonetine U1 (allthosteric site of mGluR7)

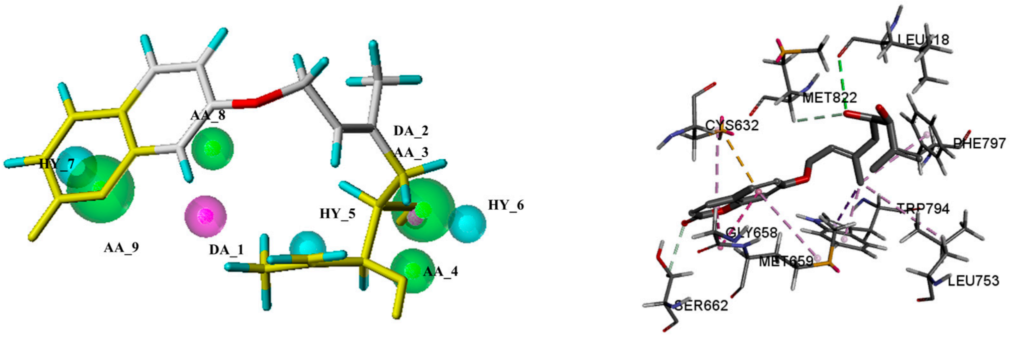

Figure S16. Pharmacophore mapping results and molecular docking results of 7-(5',6'-Dihydroxy-3',7'-dimethylocta-2',7'-dienyloxy)-coumarin (allthosteric site of mGluR8)
